# Endoscopic Collection and Analysis of Gastric Fluid DNA: A Liquid Biopsy Methodology for Tumor Biomarker Discovery

**DOI:** 10.21769/BioProtoc.5809

**Published:** 2026-09-05

**Authors:** Francine Carla Cadoná, Adriane G. Pelosof, Thais F. Bartelli, Haejin In, Lianlian Wu, Israel Tojal da Silva, Renata Pasqualini, Wadih Arap, Diana Noronha Nunes, Emmanuel Dias-Neto

**Affiliations:** 1 Masters in Health & Life Sciences, Franciscana University, Santa Maria, RS, Brazil; 2 A. C. Camargo Cancer Center, São Paulo, SP, Brazil; 3 Department of Genetics, The University of Texas, M. D. Anderson Cancer Center, Houston, TX, USA; 4 Division of Surgical Oncology, Rutgers Cancer Institute, New Brunswick, NJ, USA; 5 Early Cancer Institute, Department of Oncology, University of Cambridge, Cambridge, MA, UK; 6 Rutgers Cancer Institute, Newark, NJ, USA; 7 Division of Cancer Biology, Department of Radiation Oncology, Rutgers New Jersey Medical School, Newark, NJ, USA; 8 Division of Hematology/Oncology, Department of Medicine, Rutgers New Jersey Medical School, Newark, NJ, USA

**Keywords:** Gastric cancer, Gastric fluid DNA, gfDNA quantification, Liquid biopsy, Progression-free cancer survival, Tumor biomarker, Upper digestive tract cancer

## Abstract

Gastric cancer remains a major global health challenge, and reliable prognostic biomarkers are urgently needed to guide treatment decisions. Here, we present a simple and efficient protocol for a novel liquid biopsy approach based on quantifying gastric fluid DNA (gfDNA) collected during routine esophagogastroduodenoscopy (EGD). We have previously shown that gfDNA carries gastric cancer–derived mutations; moreover, its concentration increases with tumor progression and varies according to cancer prognosis. This empirically observed increase in gfDNA may mechanistically stem from enhanced cellular turnover, tissue disorganization, dysbiosis of the local microbiota, and/or fluctuations in immune cell infiltrates. Surprisingly, however, in patients diagnosed with gastric cancer, elevated gfDNA levels were also associated with improved survival. This paradoxical finding may be reconciled by an increased anti-tumor immune cell response in treatment-responsive gastric cancers, as well as by the contribution of non-tumoral DNA from inflammatory processes within the microenvironment of the stomach. Here, we detail a standardized protocol for gastric fluid collection and processing, designed to support downstream gfDNA quantification among other potential molecular applications.

Key features

• A novel liquid biopsy approach for discovering potential biomarkers in human gastric cancer.

• Translation of simple gfDNA concentration analysis for the diagnosis and prognosis of gastric cancer.

• gfDNA cutoff metrics that discriminate between groups and enable tumor prognostic classification.

• gfDNA as a source for downstream profiling of genetic mutations and microbiome-related studies.

## Graphical overview


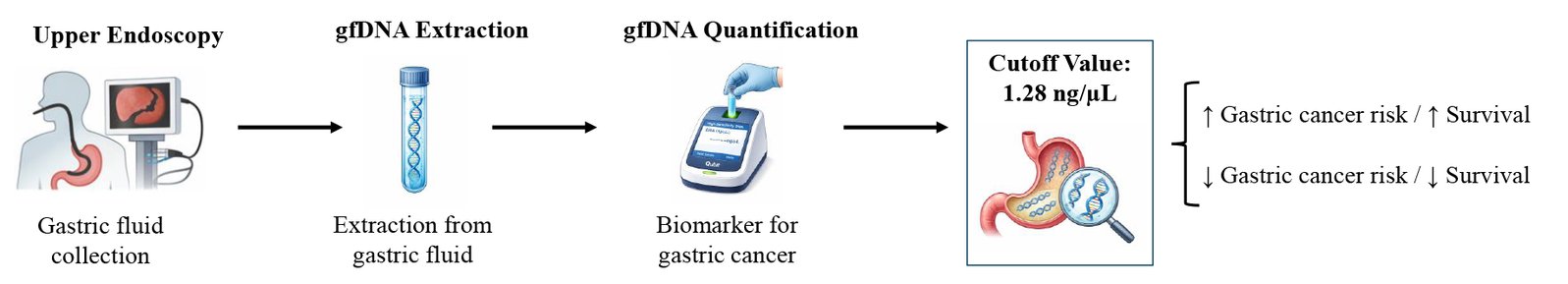


**Graphical overview of the protocol.** During routine esophagogastroduodenoscopy (EGD), gastric fluid is typically aspirated out to allow better visualization of the gastric mucosa. Instead of being incidentally discarded, the gastric fluid is used for a liquid biopsy consisting of gastric fluid DNA (gfDNA) extraction and quantification. Empirically, the gfDNA concentration increases as gastric tumors progress. Paradoxically, in patients diagnosed with biopsy-proven gastric cancer, increased gfDNA concentrations positively correlate with longer tumor progression-free survival. Establishment of cutoff values according to DNA extraction and quantification equipment and methodology standardization is recommended.

## Background

Human gastric cancer is one of the most common malignancies; worldwide, it is the fifth most diagnosed tumor type and the fifth leading cause of cancer death [1]. Esophagogastroduodenoscopy (EGD) is routinely performed to diagnose and monitor gastric cancer, perform surveillance on individuals/populations at higher risk, or to examine the upper digestive tract of those with gastrointestinal symptoms. During EGD, it is possible to determine *Helicobacter pylori* status, and tissue biopsies are collected for the pathological investigation of suspect lesions that ultimately allow gastric cancer diagnosis [2,3]. Besides previous studies that reported the identification of miRNA and proteome biomarkers of gastric cancer [4], as well as tumor-derived mutations in gastric fluid [5] and volatiles useful for cancer diagnosis [6], these fluids are usually aspirated—to allow better gastric mucosa visualization—and promptly discarded. However, the gastric fluid is enriched in biomarkers due to its close contact with the stomach mucosa and, especially, because the 8–12-h fasting period before endoscopy minimizes contamination from food residues. This improves sample quality and enables more reliable molecular analyses that provide relevant insights into gastric benign conditions and stomach cancer.

In a previous work [7], we collected gastric fluids from a large number of human subjects. In a follow-up study of this material [8], we reported a new biomarker found in gastric fluid collected during EGD examination, namely, the increased concentration of DNA found in the gastric fluid, henceforth designated as gfDNA [8]. We have shown that gfDNA can be extracted, quantified, used to support diagnosis, and, most importantly, to determine the prognosis of gastric cancer patients. To achieve this goal, we investigated the gfDNA concentration recovered from a large, unselected patient cohort (n = 1,056 patients), including human subjects with normal gastric mucosa or peptic diseases, as well as preneoplastic conditions and cancer in different stages. Several key variables were recorded, such as sex, gastric fluid pH, previous or current use of proton-pump inhibitors, tumor subtype, clinical stage, and patient outcomes.

Our studies revealed that the simple gfDNA concentration is a relevant biomarker for gastric cancer. We observed a statistically significant difference when gfDNA values were compared between subjects without gastric cancer (i.e., either normal mucosa or non-malignant peptic ulcer diseases; mean gfDNA = 10.77 ng/μL, 95% CI: 9.23–12.33; n = 606) and patients with gastric cancer at different stages (mean gfDNA = 26.86 ng/μL, 95% CI: 20.05–33.79; n = 236), with an extremely significant p-value (9.6 × 10^-11^). Furthermore, gfDNA concentrations were significantly higher when comparing lower tumor stages (T0 to T2: 15.12 ng/μL; 95% CI: 9.73–20.50) with more advanced disease (T3: 25.66 ng/μL; 95% CI: 19.46–31.85; n = 91; p = 5.97 × 10^-4^). Additionally, patients with preneoplastic conditions exhibited significantly lower gfDNA levels (mean = 10.10 ng/μL; 95% CI: 7.59–12.60; n = 99) compared to gastric cancer cases (p = 1.1 × 10^-5^). In contrast, gfDNA concentrations did not differ between the non-cancer groups, including potentially preneoplastic conditions, and subjects without biopsy-proven cancer (p = 0.89).

We integrated a protocol using standard clinical kits and equipment into routine clinical practice and identified an optimal DNA concentration cutoff via maximally selected rank statistics (maxstat.test; see Software and databases section), based on the adjusted log-rank test [9]. Gastric cancer patients with gfDNA values ≥1.28 ng/μL had better cancer prognosis and improved patient recurrence-free survival. This empirical finding may perhaps be explained mechanistically by the observed correlation between gfDNA and increased immune cell infiltration into tumors.

Notably, the protocol presented here exploits incidental material that would otherwise be discarded during EGD, thereby providing both diagnostic support and prognostic assessment of human gastric cancer at no additional collection costs. Of course, this protocol does not aim to replace EGD, which remains indispensable for diagnosis and surveillance; rather, it seeks to complement the procedure and add value by leveraging otherwise routinely discarded material for ancillary biomarker discovery and/or analysis. The samples collected may have further translational applications, including the determination of tumor-specific methylation profiles and mutations, precision medicine applications, fragmentomics, and microbiome studies. Future applications could involve analysis of the transcriptome of gastric fluid—including the promising evaluation of deconvolution studies to determine the immune profile—as well as assessment of its prognostic value in other upper digestive tract tumors, such as tumors of the gastroesophageal junction and of the esophagus. Given its potential for experimental discovery and utilization of new tumor biomarkers for cancer of the stomach—which remains largely a current unmet need—we hope that this protocol will encourage the collection and investigation of gastric fluids. If confirmed, liquid biopsy of gastric fluid may offer a readily accessible source of potentially informative biomarkers for upper digestive tract tumors, which could ultimately advance the translational study and clinical management of these malignancies.

## Materials and reagents

**Biological materials**

1. Gastric fluid and gastric fluid DNA (gfDNA) collected from patients undergoing upper endoscopic examinations

**Reagents**

1. pH measuring stripes (e.g., Sigma-Aldrich, catalog number: P4786-100EA)

2. Sodium hydroxide, 1 N solution (Sigma-Aldrich, catalog number: S2770)

3. Cell lysis solution (QIAGEN, Gentra Puregene Blood Kit, catalog number: 158467)

4. Proteinase K (Sigma-Aldrich, catalog number: 1.24568)

5. Phenol:chloroform:isoamyl alcohol (25:24:1) (Sigma-Aldrich, catalog number: P2069)

6. Chloroform (Sigma-Aldrich, catalog number: C2432)

7. Ethanol 100% (Merck, CAS 64-17-5)

8. Ethanol 70% (prepared in molecular biology–grade water) (Merck, CAS 64-17-5)

9. Sodium acetate (prepared in molecular biology–grade water) (Sigma-Aldrich, catalog number: S2889)

10. Water, molecular biology grade (Thermo Fisher Scientific, catalog number: 10977-015)

11. Qubit^TM^ dsDNA High Sensitivity Assay kit (Thermo Fisher Scientific, catalog number: Q32854)

**Solutions**

1. Sodium acetate (see Recipes)

**Recipes**

**1. Sodium acetate, 3 M, pH 5.2**

**Table:** 

Reagent	Final concentration	Volume (1 L)
Sodium acetate (anhydrous, MW = 82.03 g/mol)	3 M	246.1 g
Molecular biology–grade double-deionized water	-	q.s. to 1,000 mL

Filter sterilize (0.22 μm). Store at room temperature (RT) for 7 days or at 4 °C for up to 12 months.

**Laboratory supplies**

1. Microcentrifuge tubes, 2.0 mL, DNase/RNase-free

2. Microcentrifuge tubes, 1.5 mL, DNase/RNase-free

3. Sterile pipette tips (10, 200, 1,000 μL)

4. Serological pipettes (5 and 10 mL, sterile)

5. Cryovials (DNase/RNase-free)

6. Cryoboxes

7. Glass beakers (≥500 mL)

8. Qubit^TM^ assay tubes (Thermo Fisher Scientific)

9. Disposable gloves (nitrile, powder-free)

10. Parafilm^®^ M sealing film (Heathrow Scientific, or similar)

11. Micropipettes of variable volumes

## Equipment

1. Standard upper gastrointestinal endoscope (gastroscope) and ancillary clinical equipment for visualization, air insufflation, water irrigation, and suction/vacuum system, as available

2. -20 °C freezer

3. -80 °C ultra-low freezer

4. Microcentrifuge (refrigerated)

5. Vortex mixer

6. Thermoblock

7. Water bath

8. Qubit^TM^ Fluorometer (Thermo Fisher Scientific)

## Software and datasets

1. Microsoft Excel (Microsoft, https://products.office.com/en-us/excel) or similar, for general data organization

2. R software (https://www.R-project.org/), including the maxstat package (maxstat.test function) for statistical analyses

## Procedure

**Figure 1.**
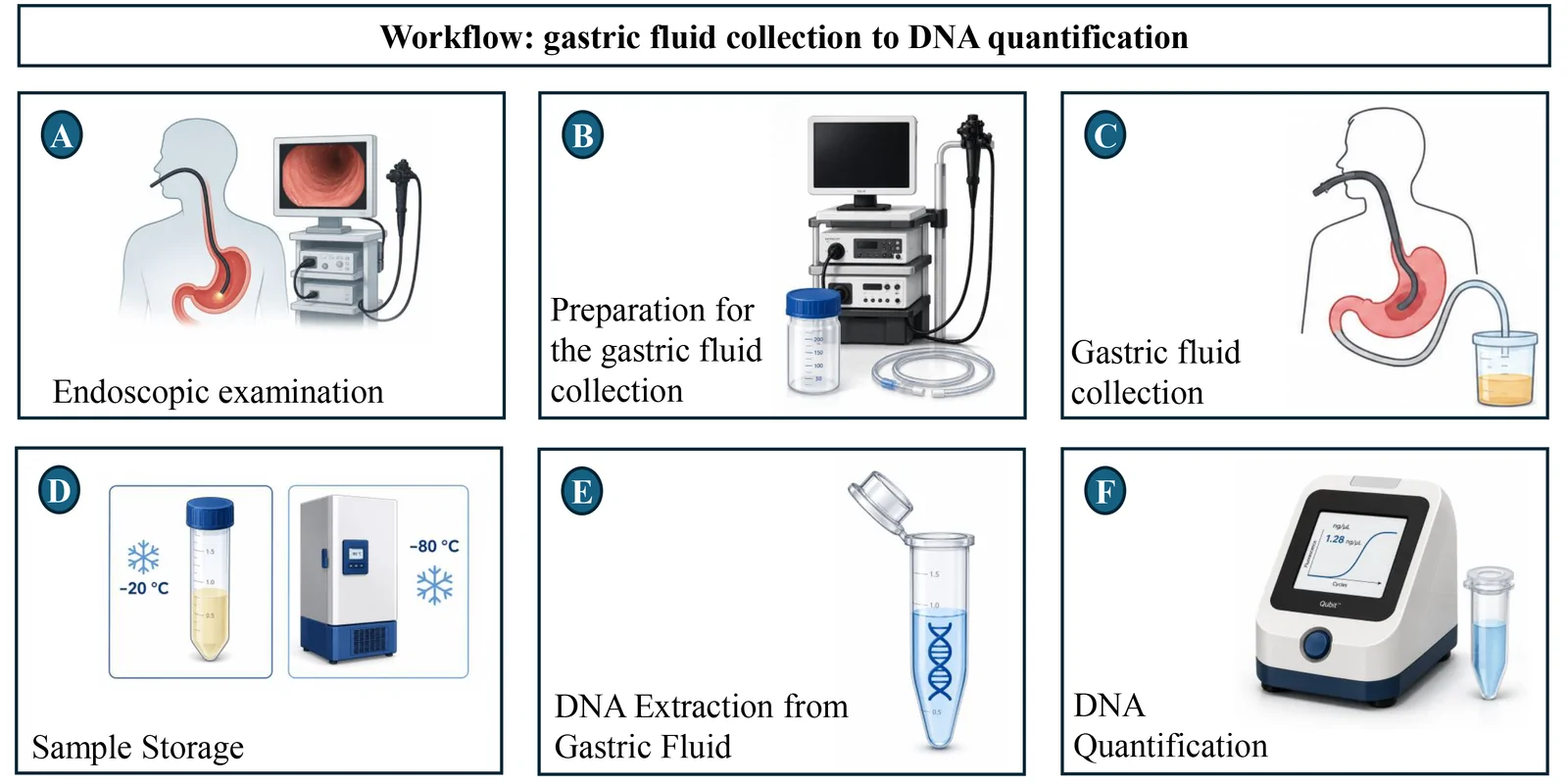
Workflow of the protocol from gastric fluid collection to DNA quantification. (A) Endoscopic examination. (B) Preparation for gastric fluid collection. (C) Gastric fluid collection. (D) Sample storage at -20 °C followed by long-term storage at -80 °C. (E) DNA extraction from gastric fluid. (F) DNA quantification.

An overview of the entire protocol workflow is shown in **Figure 1**.

**A. Endoscopic examination**

As usual and customary for upper endoscopic examination, all subjects were fasting for 8–12 h. Best-practice EGD requires a minimum examination time of 7 min, adequate gastric distension, and a systematic screening protocol for the stomach [10]. This includes antegrade inspection of four quadrants of the antrum, body, and middle-upper body, followed by retroflexed inspection of four quadrants of the fundus and cardia and three quadrants of the middle-upper body and incisura to eliminate blind spots. As usual, in some endoscopy services (though the routine may vary by institution), a 1-mL solution of 75 mg of simethicone (or similar anti-foam agent) is administered orally a few minutes before the procedure. Because the volume is small and the same preparation is given to all human subjects undergoing EGD, any effect on gfDNA concentration and composition is likely consistent across groups and therefore bias-free.

**B. Preparation for the gastric fluid collection**

1. The endoscope intended to be used for the collection of gastric fluid should be rinsed with distilled water:

a. Place the tip of the endoscope into a beaker containing approximately 500 mL of distilled water.

b. Use a vacuum pump to drain the distilled water from the beaker into the water collection tank.

c. Discard the water from the collection tank.

d. Rinse both the collection tank and the beaker with distilled water.

f. Operate the vacuum pump to drain the inner channel of the endoscope.

**Figure 2.**
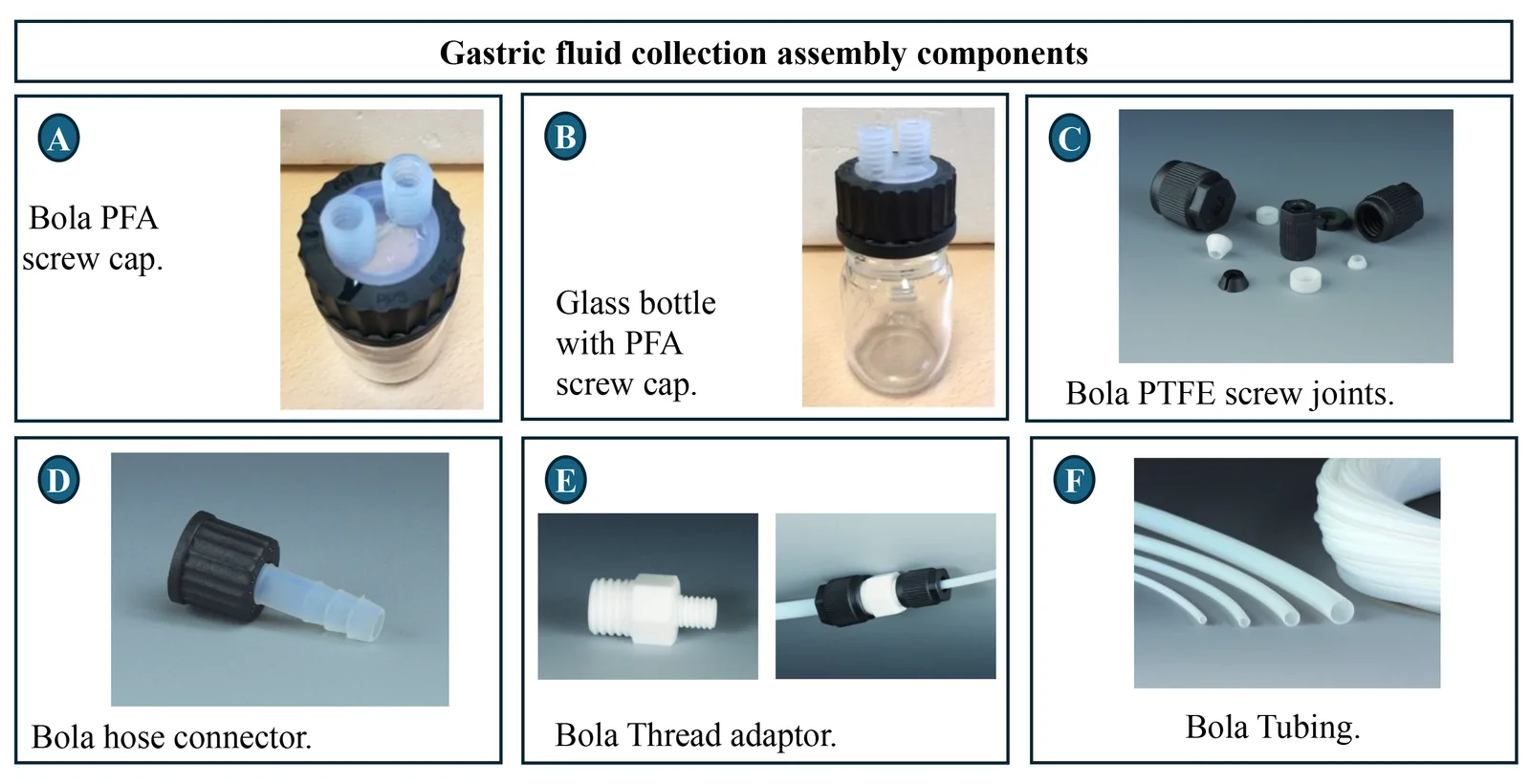
Components of the gastric fluid collection container. (A) Perfluoroalkoxy alkane (PFA) screw cap. (B) Glass bottle (250 mL). (C) Polytetrafluoroethylene (PTFE) screw joints. (D) PFA hose connector. (E) PTFE thread adaptor. (F) PTFE tubing. Examples given from connectors sold by www.bola.de.

**Figure 3.**
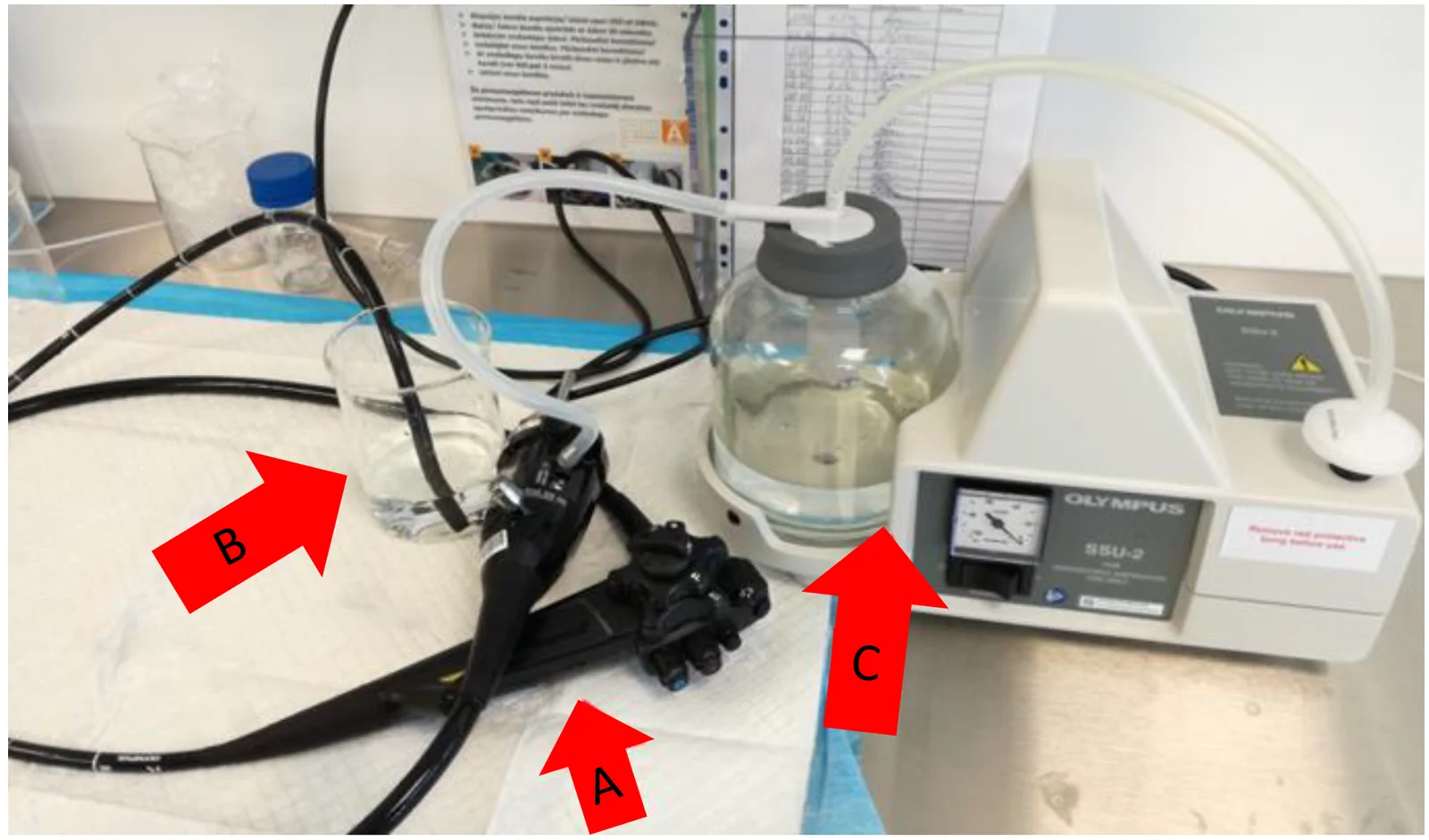
Rinsing of the internal endoscope channel with distilled water. (A) Endoscope. (B) A beaker with 500 mL of distilled water. (C) Vacuum pump with collection tank.

2. If microbiome studies are planned, collect 1 mL of the rinse water as a negative control. Samples should be initially stored at -20 °C for 3–6 h and then transferred to -80 °C for long-term storage. An overview of the containers and connections is given in **Figures 2 and 3**.

**C. Gastric fluid collection**

**Figure 4.**
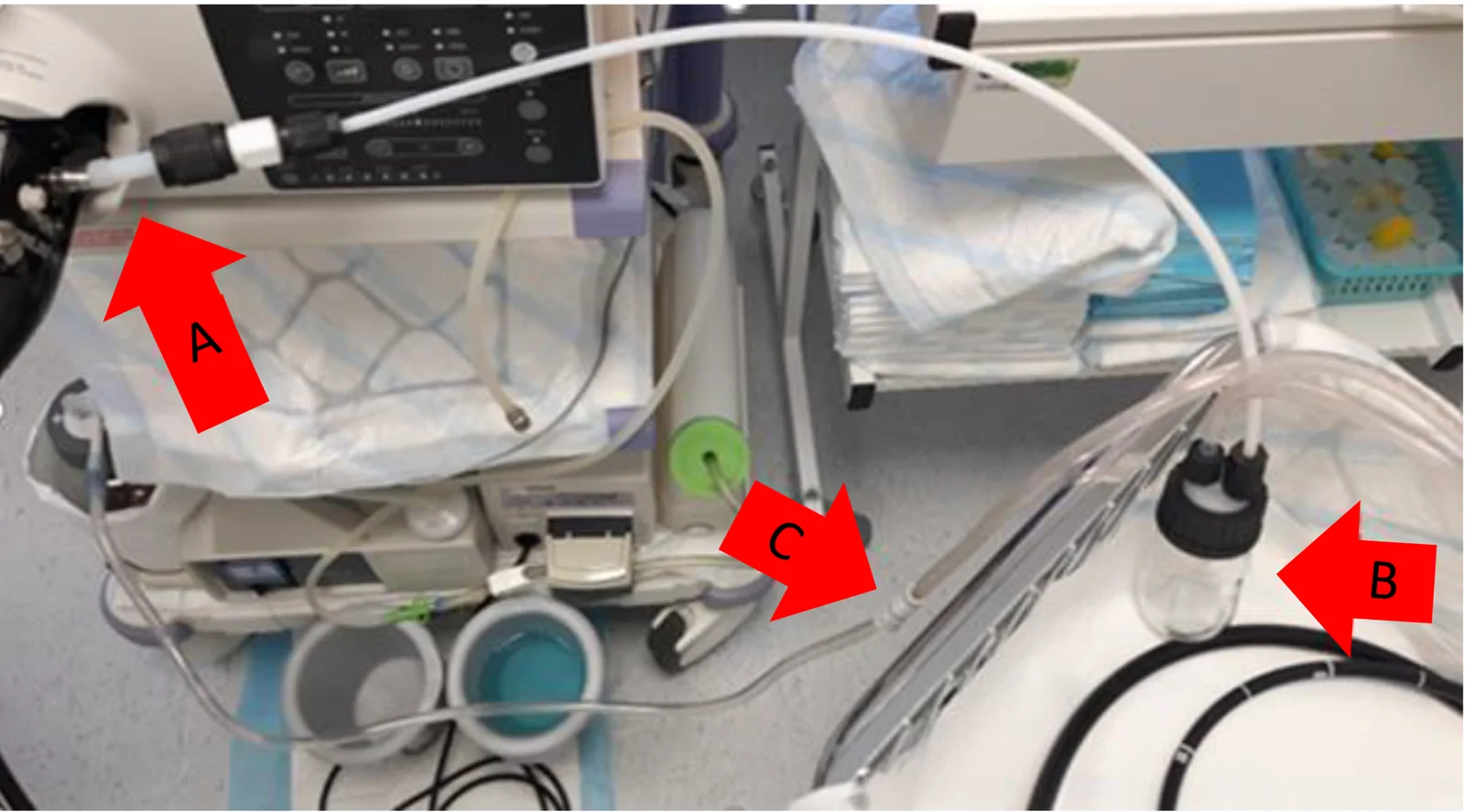
Organization of the gastric fluid collection system during endoscopy. (A) The gastric fluid collecting container is connected to the endoscope. (B) Gastric fluid collecting container. (C) The gastric fluid collecting container is connected to the vacuum pump.

1. Attach the gastric fluid collection container to the endoscope.

2. Connect the collection container to the vacuum pump.

3. At the beginning of the endoscopic examination, the operator drains gastric fluid into the collection container.

4. After collection, disconnect the container from both the endoscope and the vacuum pump. This arrangement is illustrated in **Figure 4**.

**D. Sample storage**

1. The pH of gastric fluid varies. In healthy individuals, fasting gastric fluid is typically highly acidic, with a pH of ~1.5–2.0; however, in patients with gastric diseases, especially gastric cancer, the pH may be elevated, and the fluid may be acidic, neutral, or non-acidic. Acidic conditions may lead to DNA depurination and hydrolysis. To limit these processes, immediately after collection, measure and neutralize the samples of gastric fluid pH using pH measuring stripes and carefully adding a few microliters of the 1N NaOH solution.

2. Prepare aliquots of neutralized gastric fluid samples (800 μL) in 2.0 mL tubes. The number of aliquots depends on the volume of gastric fluid recovered, the aim of the study, and freezer space available. Store tubes at -20 °C for initial freezing.

3. After 3–6 h, transfer the samples to -80 °C for long-term storage.

4. Maintain samples continuously frozen at -80 °C throughout the entire storage period.

5. As per the best practices, always avoid freeze/thaw cycles during storage and/or sample handling.

**E. DNA extraction from gastric fluid**

1. Add 500 μL of cell lysis solution to 800 μL of a gastric fluid aliquot.

2. Add 15 μL of Proteinase K (20 μg/μL).

3. Incubate at 55 °C under agitation (300 rpm) for approximately 1 h or until complete lysis is achieved (a water bath may be used). For highly viscous samples, incubation may be extended overnight (up to 18 h). Complete lysis is indicated by the absence of visible particulate material and a homogeneous lysate.

4. Add 500 μL of phenol:chloroform:isoamyl alcohol (25:24:1, v/v/v) to the lysate.

5. Vortex for 1 min until the solution becomes milky.

6. Centrifuge at ~16,000× *g* for 10 min at RT.

7. Transfer up to 950 μL of the upper aqueous phase to a new 2.0 mL tube.

8. Add 500 μL of phenol:chloroform:isoamyl alcohol (25:24:1, v/v/v).

9. Gently vortex until the solution becomes milky.

10. Centrifuge at ~16,000× *g* for 10 min at RT.

11. Add 500 μL of chloroform directly to the tube.

12. Repeat step E9.

13. Centrifuge at ~16,000× *g* for 10 min at room temperature.

14. Transfer up to 950 μL of the upper aqueous phase to a new 2.0 mL tube.

15. Add 800 μL of cold 100% ethanol and 10% volume (80 μL) of 3 M sodium acetate.

16. Mix thoroughly by inversion or brief vortexing.

17. Incubate at -20 °C for at least 1 h or overnight. This step can be skipped if a visible DNA precipitate is already present.

18. Centrifuge at ~20,000× *g* for 30 min at 4 °C.

19. Carefully remove the supernatant by inversion.

20. Add 1 mL of ice-cold 70% ethanol.

21. Gently detach the pellet from the bottom of the tube during the first wash (side-vortexing may help).

22. Centrifuge at ~20,000× *g* for 5 min at 4 °C.

23. Repeat the washing steps (E20–21).

24. Centrifuge at ~20,000× *g* for 3 min at 4 °C.

25. Remove the supernatant carefully by inversion.

26. Perform a quick spin and remove residual ethanol with a pipette.

27. Dry the pellet at room temperature or 42 °C until no ethanol remains.

28. Keep the tube open during drying.

29. Resuspend the pellet in 100 μL of nuclease-free water.

30. Homogenize with a pipette.

31. Incubate at 55 °C for 10 min in a thermoblock.

32. If the pellet is not fully dissolved, incubate overnight at 4 °C.

33. Measure DNA concentration by using a Qubit High Sensitivity DNA assay.

**Figure 5.**
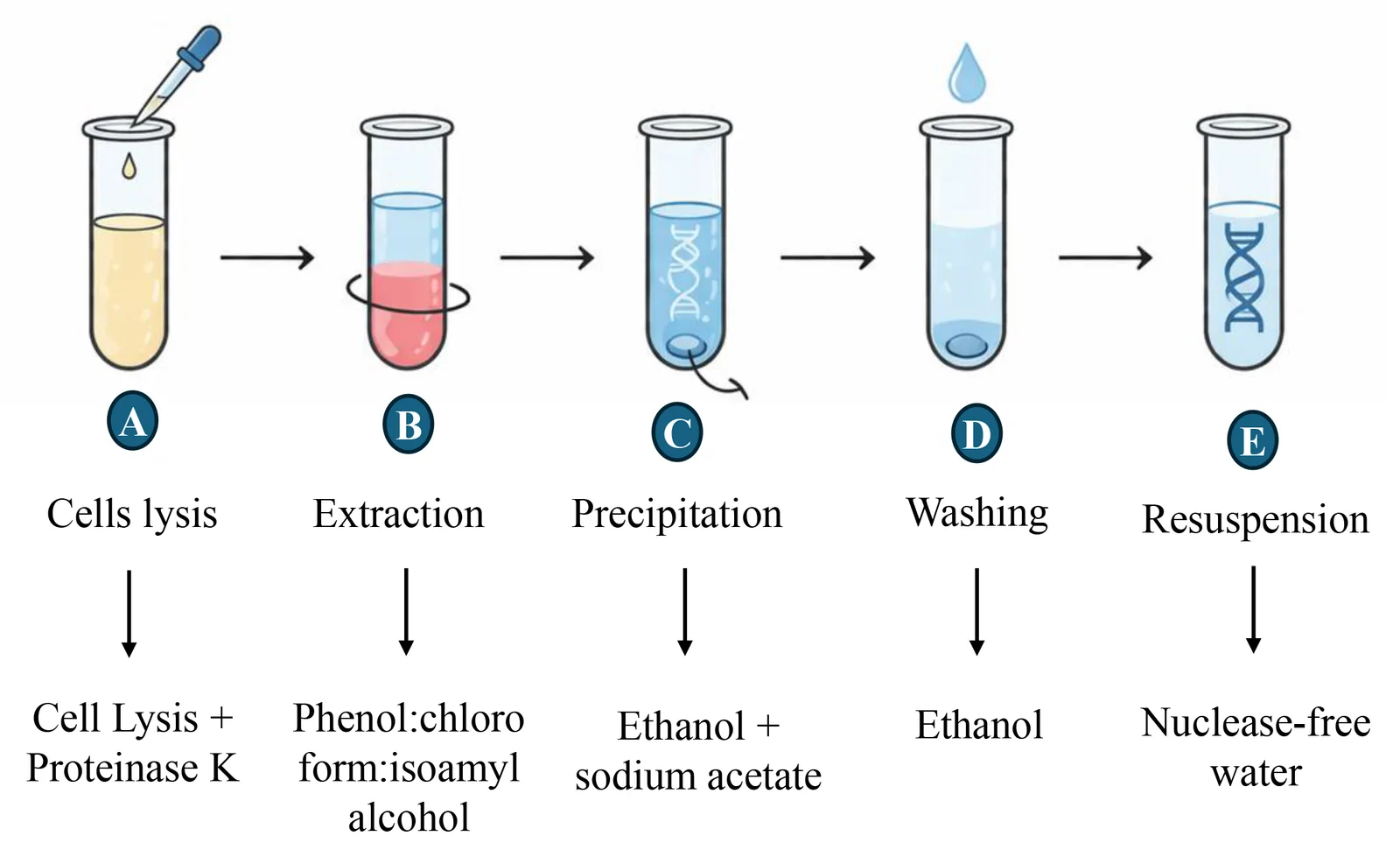
DNA extraction steps from gastric fluid

*Note: These steps are summarized in* ***Figure 5***.

**F. DNA quantification**

1. Add 198 μL of Qubit working solution to an assay tube.

2. Add 2 μL of the DNA sample.

3. Mix by gently vortexing or pipetting, avoiding the formation of bubbles.

4. Keep the samples at room temperature for 2 min before DNA quantification.

## Data analysis

1. Calculate the average of the gfDNA replicate quantification values.

2. Organize the data in Microsoft Excel and import the dataset into R.

3. Install and load the maxstat package, which contains the maxstat.test function: install.packages("maxstat") and library(maxstat).

4. Use the maxstat.test function to determine the optimal gfDNA cutoff value based on your own dataset. We recommend an independent evaluation of the results using maxstat.test, followed by the log-rank test to determine the optimal cutoff value.

5. As a reference, the gfDNA cutoff value obtained in our study to indicate prognosis (1.28 ng/μL) was derived from DNA extracted from 800 μL of gastric fluid and resuspended in 100 μL, as described in this protocol.

6. In our report [8], individuals with gfDNA concentrations >1.28 ng/μL showed a better prognosis.

## Validation of protocol

This protocol has been applied and validated in:

Cadoná et al. [8]. Endoscopic liquid biopsies of gastric fluid in a large human patient cohort reveal DNA content as a candidate tumor biomarker in gastric cancer. *eLife*. 2025 Dec 16;14:RP107103. doi: 10.7554/eLife.107103. PMID: 41400466; PMCID: PMC12707811.

## General notes and troubleshooting

**General notes**

1. Gastric fluid DNA represents total DNA available and does not distinguish between cellular origins.

2. In some settings, DNA quantity will be below the detection limit.

3. The volume of gastric fluids in patients will vary markedly. In a small fraction of patients, no gastric fluid is observed during endoscopy. In these cases, DNA for microbiome and tumor-DNA analysis can still be obtained by rinsing the stomach with 10–20 mL of saline, which is then aspirated and processed identically to routine gastric fluid. However, gfDNA concentration comparisons between rinsed and non-rinsed (“native”) gastric fluid samples are confounded by dilution and must be interpreted with great caution.

**Troubleshooting**

**Problem 1:** Low DNA quantity.

Possible cause: Insufficient starting volume of gastric fluid, which inherently contains low concentrations of shed epithelial cells and microbial DNA due to the harsh, degradative environment of the stomach.

Solutions: Increase the input volume by pooling two or more aliquots of gastric fluid and combining them prior to lysis, or alternatively, process multiple aliquots in parallel through lysis and binding steps and combine the resulting DNA during the final ethanol precipitation or elution step. Additionally, consider extending the proteinase K digestion time to improve cell lysis and maximize DNA release, particularly if the sample is viscous or contains mucin. Prolonged incubation at 56 °C with intermittent vortexing can significantly enhance yields from difficult specimens. Final DNA concentration should always consider the total volume of gastric fluids used during extraction.

**Problem 2:** Inconsistent quantification (high variability between replicate readings).

Possible causes: Pipetting errors, including inaccurate delivery volumes, failure to mix samples thoroughly before aliquoting, or using tips that are not properly calibrated or pre-wetted for viscous solutions. Other contributing factors may include incomplete dissolution of the DNA pellet, residual ethanol or salts interfering with absorbance measurements, or using an improperly blanked spectrophotometer.

Solutions: Ensure all samples are fully resuspended by incubating at 55 °C for 5–10 min with intermittent vortexing and brief centrifugation to collect the liquid at the bottom of the tube. Always measure each sample in technical triplicates from independent aliquots and record the average while discarding any outlier that deviates more than 10% from the mean. Use the average value for subsequent analyses. Additionally, verify that the blank is measured using the identical buffer or water used for resuspension. If variability persists, check pipette calibration and replace tips with low-retention versions to ensure consistent delivery.

**Problem 3:** Degradation during storage.

Possible causes: Delayed freezing or repeated freeze/thaw cycles.

Solutions: To preserve sample integrity, gastric fluid should be processed or frozen as rapidly as possible after collection—ideally within 30 min—by snap-freezing in liquid nitrogen or a dry ice–ethanol bath before transfer to storage conditions. For short-term storage (up to a few weeks), samples may be kept at -20 °C, but for long-term preservation (months to years), they should be transferred to -80 °C to minimize enzymatic activity and nucleic acid hydrolysis. To completely avoid repeated freeze/thaw cycles, divide the sample into single-use aliquots immediately upon collection, ensuring that each aliquot contains sufficient volume for one complete extraction and that aliquots are not refrozen once thawed. Additionally, consider adding a nuclease inhibitor (e.g., EDTA to a final concentration of 10 mM) or a commercial preservation buffer (e.g., RNAlater or DNA/RNA Shield) prior to freezing, particularly if samples will be transported or stored for extended periods before processing. When thawing aliquots for extraction, do so rapidly on ice or at 4 °C and process them promptly without prolonged exposure to room temperature.

**Problem 4:** No DNA pellet is visible after precipitation.

Possible causes: Low DNA concentration, resulting in a transparent or invisible pellet, or pellet dislodgement during removal of supernatant or ethanol wash steps.

Solutions: Do not assume the DNA is lost; proceed with the 70% ethanol wash as usual, even if no pellet is visible. When removing supernatant, mark the side of the tube where the pellet should be (the outer side of the centrifuge rotor) and pipette slowly from the opposite side to avoid disturbing an invisible pellet. Add the 70% ethanol gently down the side of the tube without vortexing or vigorous pipetting to prevent dislodging the pellet. After washing and drying, resuspend the DNA in the smallest practical volume (e.g., 10–20 μL) of nuclease-free water.

**Problem 5:** DNA contamination with phenol or ethanol (evidenced by abnormal A_260_/A_280_ or A_260_/A_230_ ratios).

Possible cause: Incomplete removal of the organic phase during phenol–chloroform extraction.

Solutions: Carefully remove the upper aqueous phase without disturbing the interphase or lower organic layer—using a fine-tipped pipette and leaving a small margin of safety (e.g., 10–20 μL of aqueous phase behind) is preferable to inadvertently transferring phenol. Performing a second chloroform extraction (without phenol) can help eliminate any residual phenol carried over from the first extraction. For ethanol contamination, after the 70% ethanol wash, remove as much supernatant as possible using a fine pipette, then air-dry the pellet at room temperature for 10–20 min until no visible moisture remains and the pellet appears slightly translucent or glassy; alternatively, use a speed-vac or gentle heating at 37 °C for 5–10 min to expedite drying, but avoid overdrying as this makes DNA difficult to redissolve. Also, measure the A_260/230_ ratio, as values below 1.8 indicate phenol or ethanol carryover, while values above 2.2 may suggest residual ethanol.
